# Latent profiles of childhood psychological maltreatment and their links to adult mental health in China and the UK

**DOI:** 10.1186/s13034-023-00572-4

**Published:** 2023-02-24

**Authors:** Zhuoni Xiao, Ingrid Obsuth, Franziska Meinck, Aja Louise Murray

**Affiliations:** 1 Department of Psychology, University of Edinburgh, 7 George Square, Edinburgh, EH8 9JZ UK; 2 Clinical & Health Psychology, University of Edinburgh, Edinburgh, UK; 3 School of Social and Political Science, University of Edinburgh, Edinburgh, UK; 4 Faculty of Health Sciences, North-West University, Vanderbijlpark, South Africa; 5 School of Public Health, University of the Witwatersrand, Johannesburg, South Africa

**Keywords:** Latent profile analysis, Psychological maltreatment, Mental health, Cultural differences, Mental well-being

## Abstract

**Background:**

Though links between childhood maltreatment and mental health have been established, little known about how specific types of childhood maltreatment tend to cluster and how the resulting patterns of exposure impact mental health outcomes.

**Method:**

The current study used latent profile analyses in Chinese (N = 544) and UK (N = 589) samples to identify childhood psychological maltreatment profiles (i.e., profiles of psychological abuse, psychological neglect, and psychological non-support) in different country contexts, and their associations with a range of mental health (i.e., depression, anxiety, anger, physical aggression, verbal aggression, and hostility), and broader well-being (i.e., self-esteem) outcomes. Unadjusted as well as analyses adjusted for adverse childhood experiences (ACEs) were conducted.

**Results:**

Four profiles were identified in both samples, but their nature differed between the Chinese sample (“*Psychological Non-support*”, “*Low-Maltreated*”, “*High-Maltreated*”, and “*Severe-Maltreated*”) and the UK sample (“*Low-Maltreated*”, “*Moderate-Maltreated*”, “*High-Maltreated*”, and “*Severe-Maltreated*”). Individuals in the “*Psychological Non-support*” in China and “*Low-Maltreated*” class in the UK displayed better mental health outcomes–lower levels of depression, anxiety, and aggression, and higher self-esteem. In contrast, individuals in the “*Severe-Maltreated*” profiles in both the Chinese and UK samples displayed poorer mental health outcomes–higher depression, anxiety, and aggression, and lower self-esteem. Interventions and prevention efforts are needed for individuals categorized in profiles affected by psychological maltreatment.

**Conclusion:**

This study highlights the importance of using targeted intervention or prevention to prevent psychological maltreatment, as well as improve mental health outcomes in individuals who have experienced psychological maltreatment.

**Supplementary Information:**

The online version contains supplementary material available at 10.1186/s13034-023-00572-4.

## Introduction

Childhood maltreatment is common worldwide, with an estimated prevalence of sexual abuse of approximately 2.6–12.8%; physical abuse of approximately 6.7–18.9%; psychological abuse of approximately 9.2–33.4%; and neglect of approximately 6.6–47.2% [43]. It has negative impacts on a wide range of mental health and behavioural outcomes, such as depression and anxiety [30], suicidality [4], and violence [27] [65, 66]. However, a majority of past childhood maltreatment studies have focused on specific forms of childhood maltreatment and their links to mental health outcomes and there has been a relative lack of research on psychological maltreatment in particular [28, 29]. Person-centred analyses, such as latent profile analyses, allow common exposure patterns to be identified among subgroups of participants [42]. Previous studies utilizing this method have shown how the method can provide illumination on adverse childhood experiences (e.g., [21, 34],see [51] for review). For example, Hemady et al. [34] conducted a latent profile analysis of adverse childhood experiences in a multi-country cohort and found that the sample could be divided into four classes (i.e., “*Highly Maltreated*”, “*Emotionally and Physically Abused with Intra-familial Violence Exposure*”, “*Emotionally Abused”,* and “*Low Household Dysfunction and Abused*”) differing in their outcomes. While these previous studies demonstrate the value of the latent class approach to illuminating abuse profiles and their consequences, only a few (e.g., [48, 62] have provided an in-depth exploration of different childhood psychological maltreatment profiles (i.e., psychological abuse, psychological neglect, and psychological non-support) specifically. This study adds to the existing literature by applying a latent profile analysis to explore latent subtypes of childhood psychological maltreatment.

Childhood psychological maltreatment refers to a repeated pattern of caregivers’ behaviours or extreme incident(s) that convey to children that they are worthless, flawed, unloved, unwanted, endangered, or of value only in meeting another’s needs [3]. It can be divided into psychological abuse (sometimes termed “emotional abuse”) and psychological neglect (sometimes termed “emotional neglect”). (In this paper, the terms psychological abuse and psychological neglect will be used throughout for consistency). Psychological abuse refers to the commission of hostile acts by caregivers towards the child (McGee and Wolfe, 1991), behaviours such as belittling, restricting social interaction, and intentionally trying to scare, humiliate, ignore, or isolate could be seen as psychological abuse. Psychological neglect refers to caregivers’ acts of omission in failing to provide necessary care for children, which may include meeting their basic needs [22], behaviours such as providing little or no warmth nurturing, or praise during any developmental period in childhood, and being detached or uninvolved, interacting only when necessary, could be seen as psychological neglect. Psychological support refers to gestures or acts of caring, acceptance, and assistance expressed by caregivers towards a child [59]. Caregivers who fail to these behaviours would be seen as providing a lack of psychological support (i.e., psychological non-support).

The latent profile subtypes that emerge from methods such as LPA are particularly valuable if they encode meaningful information about differential causes or outcomes of psychological maltreatment. For example, if the subtypes from LPA can be used to predict which mental health issues an individual is at risk of, they can provide not only a description of common patterns of psychological maltreatment but also clinically valuable information that can forecast risk and potentially inform the targeting of interventions. A wide range of mental health symptoms has been linked to psychological maltreatment [65, 66], making them key outcomes to investigate in LPA-derived latent subtypes.

Previous studies have been identified negative impacts of childhood psychological maltreatment on mental health. A systematic review [30] demonstrated significant associations between psychological abuse and depression and anxiety. Previous studies have also suggested significant associations between childhood psychological neglect and adult psychiatric disorders such as depression, anxiety, psychotic disorders, and substance abuse. For instance, Salokangas et al. [54] found that physical abuse and psychological neglect were strongly associated with adult psychiatric disorders.

Childhood psychological maltreatment has also been suggested to affect externalizing problems. According to Social Learning Theory (SLT; [9], children observe their caregivers’ behaviour to adapt their behaviour in social contexts. Vicarious learning is more likely when the model of the observer is rewarded, but it can also occur without favourable consequences. Based on this theory, some children learn to do what has been done to them (as well as what they witness). The child victim later becomes a perpetrator, creating one route to the intergenerational transmission of violence [26]. Indeed, children who have experienced physical abuse are more likely to engage in violent behaviours, while children who have experienced sexual abuse are more likely to engage in sexual offences [26]. Based on these previous empirical studies on other types of maltreatment and aggression, children who have experienced psychological abuse or psychological neglect should therefore be more likely to engage in aggressive behaviours such as verbal violence, anger, or hostility [10, 19].

Childhood psychological maltreatment is also negatively associated with broader well-being markers, such as self-esteem [19]. According to Stanley Coopersmiths’ self-esteem theory (1959), self-esteem is rooted in early childhood with a foundation of trust, unconditional love, and security, which is impacted as life progresses by a combination of positive and negative evaluations. From this perspective, childhood psychological abuse and psychological neglect represent key risk factors for poor self-esteem development. Indeed, recent empirical investigations have found evidence consistent with this claim. For instance, Badr et al. [6] found that childhood psychological maltreatment was a significant predictor of low self-esteem after adjusting for potential confounders, and Chen and Qin [13] found that childhood psychological abuse was negatively associated with self-esteem. Studies that have explored childhood psychological neglect have also suggested it has negative associations with self-esteem [45, 49].

### Current study

The objective of the present study was to examine whether LPA-derived psychological maltreatment profiles in two samples from different country contexts were associated with a range of mental health and broader well-being outcomes. We first sought to identify different sub-populations (i.e., profiles) within the samples based on their combined levels of childhood psychological abuse, psychological neglect, and psychological non-support. We then investigated the associations between these profiles and mental health outcomes (i.e., depression and anxiety), aggression (i.e., anger, physical aggression, verbal aggression, and hostility), and well-being (i.e., self-esteem).

## Methods

### Sample and procedure

A general community sampling approach was used in the present study. Participants were not specifically recruited based on their experiences of psychological maltreatment; however, the focus on psychological maltreatment was made clear in the information sheet which was provided to participants before the study. Five hundred and forty-four participants (59.9% aged 21–30; 63.4% females; 61.6% mother as primary caregiver) were recruited via social media in China. Five hundred and eighty-nine participants (60.6% aged 21–30; 63% female; 84.2% mother as primary caregiver) were recruited via the local university volunteer panel and Prolific in the UK. Participants from China and Prolific were offered 2 pounds as compensation, while participants from local university volunteer panels were offered course credits. Both groups completed an online questionnaire utilizing the Qualtrics platform. A full description of the demographic characteristics of the participants is provided in Additional file 1: Tables S1 (China) and S2 (the UK).

### Measure

*Childhood Psychological Maltreatment* was measured using the Psychological Maltreatment Review (PMR; [11], adapted from Xiao et al. (2022a, b). Three sub-factors were measured with 30 items—Psychological Abuse, Psychological Neglect, and Psychological Non-support. Participants were asked to rate their caregivers’ behaviours for each item from 0 (never) to 6 (over 20 times per year). Example item contents for psychological abuse included “*Insulted you*”, “*Criticised you*”, or “*Called you names*”; example item contents for psychological neglect included “*Act like they didn’t seem to care you*”, “*Ignore you*”, or “*Act like you weren’t there, even though you were*”. Psychological non-support (i.e., lack of psychological support) was measured by item contents such as “*Hugged you*”, “*Encouraged you to have friends*”, or “*Tried to make you better when you were upset or hurt*”. The psychological non-support item scores were reversed when calculating the sum scores. Higher scores represent a higher level of psychological abuse, neglect and non-support. As reported in our previous work [65, 66], two Psychological Non-support items of the Chinese version were removed due to evidence of cultural differences in the meaning of these items, complicating the comparison of findings across contexts.

*Depression* was measured using the Patient Health Questionnaire-9 (PHQ-9), which has been established as a reliable screening tool for depression [18, 38]. Participants were asked to indicate whether they suffered from nine symptoms over the past two weeks on 0 (not at all), 1 (several days), 2 (more than half the days), and 3 (nearly every day). Scores lower than 4 represent minimal depression, which means no need for depression treatment. Scores between 5 and 9 represent mild depression, while scores between 10 to 14 represent moderate depression. Scores between 15 and 19 represent moderately severe depression, and scores higher than 20 represent severe depression.

*Anxiety* was measured using the Clinical Anxiety Scale (CAS; [60]. Previous studies have suggested acceptable reliability [37]. In the current study, we adopted the Chinese version of CAS from our previous work [65, 66]. Participants were asked to respond to 25 items with response options from 0 (rarely none of the time) to 4 (most or all of the time). Higher scores represent higher levels of anxiety. The clinical cut-off point for this measure is a score of 30.

*Aggression* was measured by the Buss-Perry Aggression Questionnaire (BPAQ; [12]. There are four sub-scales within the BPAQ–anger (7 items), physical aggression (9 items), verbal aggression (5 items), and hostility (8 items). All the subscales were used for outcome variables to explore the associations between different profiles and dimensions of aggression. Allen and Anderson [2] pointed out the meaningful distinctions between different forms of aggression which have been identified, necessitating the use of separate subscales to capture all these aggression concepts. A five-point Likert scale from ‘extremely uncharacteristic of me’ to ‘extremely characteristic of me’ was used. Previous studies have shown the high reliability of the BPAQ in a different context [55].

*Self-esteem* was measured using the Rosenberg Self-Esteem Scale [53]. Participants were asked to respond to 10 items using a four-point scale from ‘strongly disagree’ to ‘strongly agree’. Higher scores represent a higher level of self-esteem. A score between 20 and 30 is within the normal range. Scores below 20 mean low self-esteem. The Rosenberg self-esteem scale has been widely used in a different context, with evidence for acceptable reliability [52, 64].

*Adverse Childhood Experience *as a covariate was measured using Adverse Childhood Experiences (ACE, developed by the Centre for Disease Control and Prevention, US; [25]. The ACE has been widely used across different ethnic groups [20, 33]. Ten items using dichotomous response options (i.e., yes or no) covered three dimensions—childhood abuse, neglect, and household dysfunction. We removed items 1 and 4 as they measured psychological abuse and psychological neglect in our data analysis.

Cronbach’s alpha for each measure is presented in Additional file 1: Table S3.

### Data analysis

First, latent profile analysis (LPA) was used to identify subgroups of participants with similar patterns across three types of childhood psychological maltreatment when controlled for ACE. These models were implemented in *Mplus* 8.4 using the robust maximum likelihood estimator (MLR). Solutions with one to eight profiles were examined to determine an optimal profile model. The optimal model was selected based on criteria including Akaike’s Information Criteria (AIC; [1], Bayesian Information Criteria (BIC,[57], adjusted Bayesian Information Criteria (a-BIC, [58], Lo-Mendell-Rubin likelihood ratio test (LMR test; [41], where a significant LMR *p-value* indicates that a model with k number of profiles is preferred over (significantly improves upon) a model with K-1 profiles. The means of psychological abuse, psychological neglect, and psychological non-support were freely estimated across all profiles, and their variances were constrained to equality. After identifying the optimal profile groups, participants were assigned to their most likely profile groups.Second, the outcomes (i.e., depression, anxiety, anger, physical aggression, verbal aggression, hostility, and self-esteem) were investigated via the manual three step BCH approach (i.e., step 1: latent profile model was estimated using only latent profile indicator variables (i.e., psychological abuse, psychological neglect, and psychological non-support both with and without ACEs as a covariate); step 2: the most likely profile variable was created using the latent profile posterior distribution obtained during the first step; step 3: the most likely profile regressed on predictor variables (i.e., depression, anxiety, anger, physical aggression, hostility, verbal aggression, and self-esteem) taking into account the misclassification in the second step) procedure available in *Mplus* [5]. We used the ‘BCH’ method (i.e., which used a weighted multiple group analysis in step 3, where the groups correspond to the latent profiles, and thus the profile shift is not possible because the profiles are known) that allowed us to compare the means of each outcome across latent profiles and thus explore whether these outcomes were significantly different for different patterns of psychological maltreatment (Bakk & Vermunt, 2006). Because the factor structures of childhood psychological maltreatment differed in China and the UK samples [65, 66] and to leave open the possibility for broader contextual differences to emerge, we conducted the data analyses separately for these samples.

### Sensitivity analysis

LPA was used to identify subgroups of participants with similar patterns across three types of childhood psychological maltreatment without controlled for ACE. Results and interpretation were provided in Additional file 1.

## Result

### Descriptive statistics

Table 1 presents the descriptive statistics for the Chinese and UK population. The Additional file 1 presents the correlations between variables in Additional file 1: Tables S4 (China) and S5.

**Table 1:** Descriptive statistics

	China	UK	
	Mean (SD)	Mean (SD)	t (df)
PA	18.89 (13.50)	16.97 (13.67)	2.38 (1131)
PN	15.06 (12.48)	12.33 (13.73)	3.49 (1131)
PNS	18.22 (11.38)	20.81 (7.77)	− 4.51 (1131)***
ACE	0.60 (1.23)	1.14 (1.34)	− 6.99 (1101)**
Self-esteem	29.46 (5.19)	28.21 (5.12)	3.94 (1062)
Anxiety	30.75 (15.14)	30.57 (18.18)	0.18 (1084)***
Anger	18.55 (5.13)	16.19 (5.73)	6.99 (1036)**
Physical Aggression	22.87 (5.73)	18.28 (6.74)	11.75 (1036)***
Hostility	22.79 (5.92)	22.42 (6.46)	0.94 (1036)*
Verbal Aggression	14.87 (3.22)	14.17 (4.34)	2.93 (1036)***
Depression	7.07 (5.10)	10.27 (6.62)	− 8.67 (1060)***

*PA* psychological Abuse, *PN* psychological Neglect, *PNS* psychological non-support, *ACE* adverse childhood experiences

^***^p < .001

^**^p < .01

^*^p < .05

### Latent profile solution–Chinese samples

A series of latent profile models with one to eight were specified and estimated. The LMR test had a non-significant value for the 5-class model (*p* > 0.05), suggesting a 4-class optimal model. Additional file 1: Table S6 indicated that the entropy of the four-profile solution was 0.860, which is considered satisfactory [44]. Additionally, the average latent class probabilities for the four-profile model were 0.950, 0.932, 0.957, and 0.924, above the cut-off criterion of 0.80 [47]. Accordingly, we adopted the four-profile model for the Chinese samples as the best solution based on theoretical and statistical considerations.

**Fig. 1:**
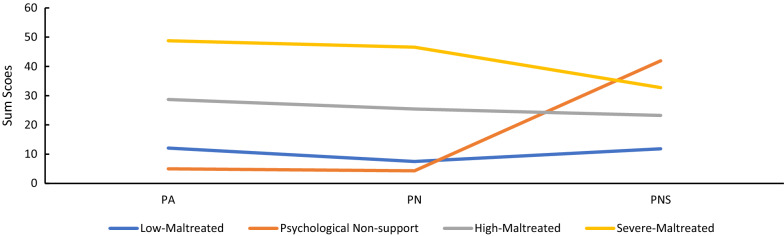
Latent profile of childhood psychological maltreatment in chinese samples. *PA* psychological abuse, *PN* Psychological neglect, *PNS* Psychological non-support. Four latent profiles were presented in the figure, “Low-Maltreated”, “Psychological Non-support”, “High-Maltreated”, and “Severe-Maltreated”, respectively

Figure 1 displays the four-profile solution that was retained. Values on the Y-axis represented the sum scores of different types of childhood psychological maltreatment. The first latent profile was the smallest and described the 5.4% of the sample who reported the lowest level of psychological abuse and psychological neglect but the highest level of psychological non-support and was thus labelled the “*Psychological Non-support*” profile. The second latent profile represented 56% of the samples labelled “*Low-Maltreated*,” given that the level of psychological abuse and psychological neglect was higher than the “*Psychological Non-support*” group but with the lowest level of psychological non-support. The third latent profile, named the “*High-Maltreated*” profile, characterised 32.9% of the sample presenting experiences of a higher level of psychological abuse, psychological neglect, and psychological non-support. The fourth latent profile, labelled “*Severe*-*Maltreated*”, includes 5.5% of the sample and reported the highest level of psychological abuse, neglect, and non-support. Additional file 1: Table S8 presents the means and standard errors of each psychological maltreatment type.

### Latent profile solution–UK samples

The four-class solution was also considered the best-fitting model for the UK sample based on a series of fit indices (see Additional file 1: Table S7 in Supplementary Material). The LMR test had a non-significant value for the 5-class model (p > 0.05), suggesting a 4-class optimal model and the BIC, which has been identified as the most reliable of the available fit indices [47], was lower than other models with significant *p*-values. We, therefore, adopted the four-profile model as the best solution for further analyses.

**Fig. 2:**
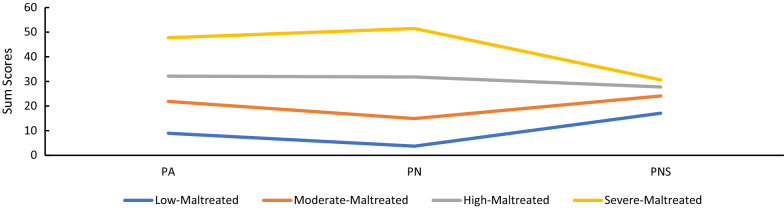
Latent profile of childhood psychological maltreatment in the UK Samples. PA psychological abuse, *PN* psychological neglect, *PNS* Psychological non-support. Four latent profiles were presented in the figure, “Low-Maltreated”, “Moderate-Maltreated”, “High-Maltreated”, and “Severe-Maltreated”, respectively

Figure 2 displays the four-profile solution for the UK sample. Values on the Y-axis represent the sum scores of different types of childhood psychological maltreatment. The first latent profile was the largest and described 57.9% of the sample who reported the lowest level of childhood psychological maltreatment and were thus “*Low-Maltreated*”. The second latent profile represented by 25.4% of the sample was labelled "*Moderate-Maltreated*," given that samples experienced moderate childhood psychological maltreatment. The third latent profile, named the “*High-Maltreated*” profile, characterised 11.2% of samples, representing a higher level of childhood psychological maltreatment. The fourth latent profile, labelled “Severe-*Maltreated*”, included 5.5% of the sample who reported the highest level of childhood psychological maltreatment. Additional file 1: Table S9 presents the means and standard errors for each form of psychological maltreatment.

### The associations between profiles and mental health—Chinese samples

**Fig. 3:**
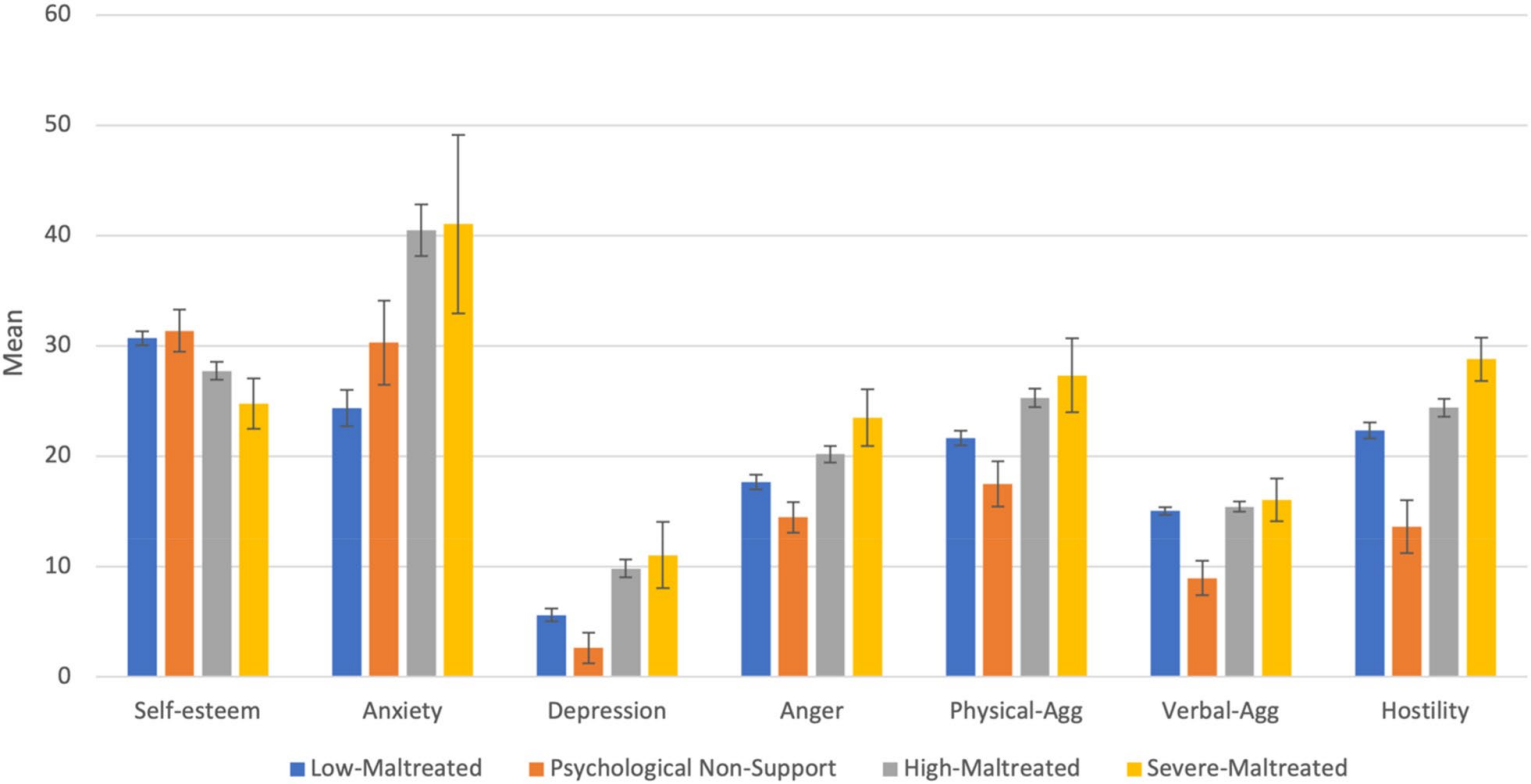
The outcomes for each profile (China). *Physical-Agg* physical aggression. *Verbal-Agg* verbal aggression. The figure presented the mean of each variable in each profile

The relationships between four latent profiles and mental health outcomes for the Chinese sample are displayed in Additional file 1: Table S10, and Fig. 3 presents the outcomes for each profile. The results indicated that the sample in the “*Psychological Non-support*” profile exhibits better mental health outcomes with the lowest depression and aggression levels. Moreover, the sample in the “Low-Maltreated” exhibit the lowest level of anxiety with the highest level of self-esteem. In addition, those assigned to the “*Severe-Maltreated*” profile show poorer mental health outcomes than other profiles, with the highest levels of depression, anxiety, and aggression and the lowest levels of self-esteem. The “*Psychological Non-support*” profile was categorised as having minimal depression. The other three profiles were associated with mild or moderate depression. The differences in levels of depression between all profiles were significant, except for the difference between the “*Moderate-Maltreated*” and “*Severe-Maltreated*” profiles. When compared to anxiety levels, the “Low-Maltreated” profile displayed the lowest level of anxiety, while other profiles were all above the clinical cut-off point. There was no difference between the “*High-Maltreated*” and “*Severe-Maltreated*” profiles, but differences between other profiles were significant.

Only the “*Psychological Non-support*” profile was below the norm mean score compared to anger. The difference between all profiles was significant. When compared to physical aggression scores, “*Psychological Non-support*” and “*Low-Maltreated*” profiles were below the norm mean score, while “*High-Maltreated*” and “*Severe*-*Maltreated*” profiles were above the norm mean scores. The differences between all profiles were significant, except for the difference between “*Moderate-Maltreated*” and “*Severe-Maltreated*”. Compared to verbal aggression scores, only the “*Psychological Non-support*” profile was below the norm mean score. The differences between the “*Low-Maltreated*”. “*Moderate-Maltreated*” and “*Severe-Maltreated*” were non-significant, while the differences between “*Psychological Non-support*” with other profiles were significant. Only the “*Psychological Non-support*” profile was below the norm mean score compared to the hostility scores. Other profiles were all above the mean scores. All the profiles showed significant differences compared with other profiles.

When compared the self-esteem levels, samples in the “*Low-Maltreated*” profiles showed a higher level of self-esteem, while other profiles were within the normal ranges. There was no difference between the “*Psychological Non-support*” and “*Low-Maltreated*” profiles; however, the differences between other profiles were significant.

### The associations between profiles and mental health—UK samples

**Fig. 4:**
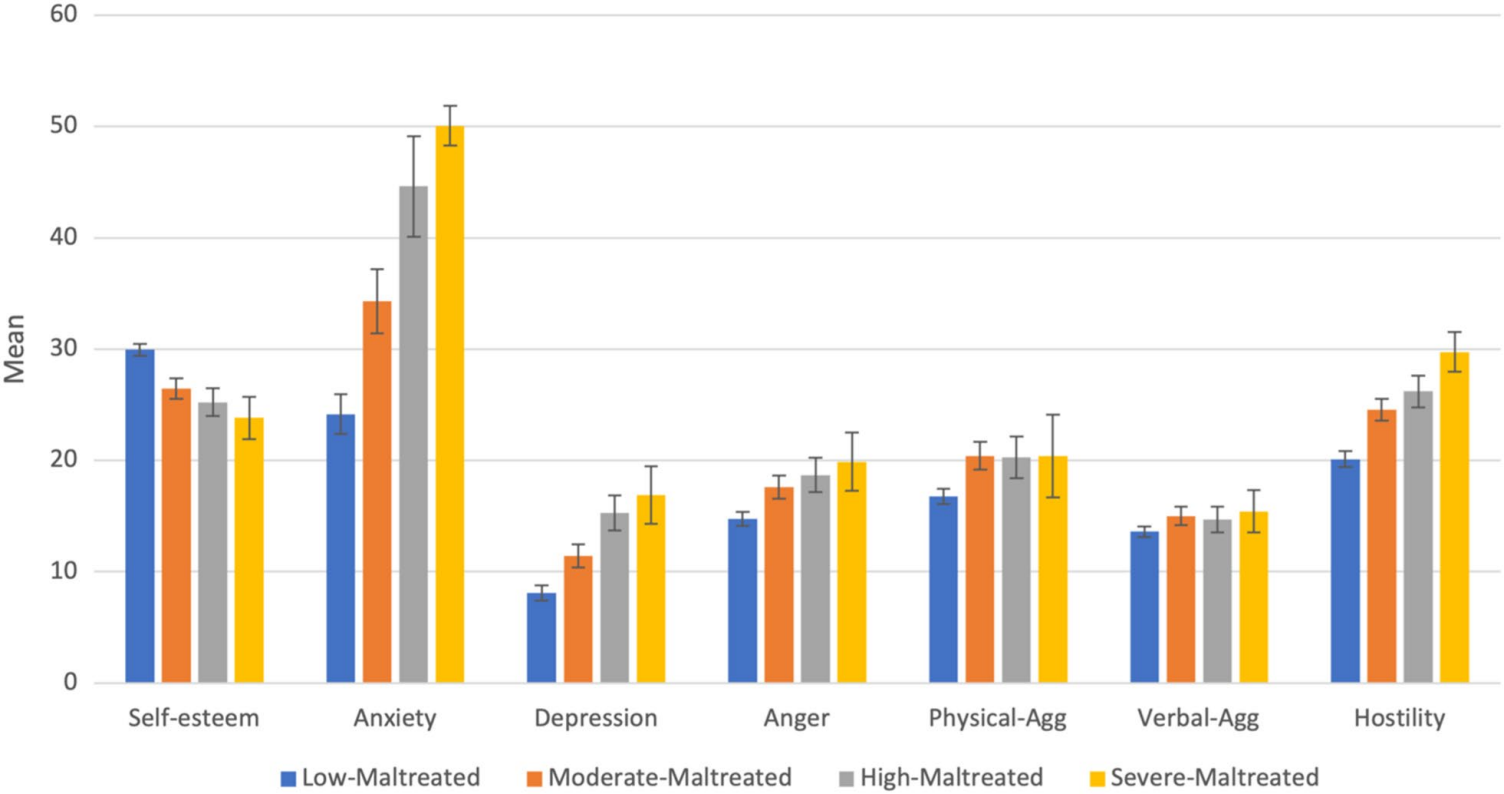
The outcomes for each profile (UK). *Physical-Agg* physical aggression, *Verbal-Agg* verbal aggression. The figure presented the mean of each variable in each profile

The relationships between four latent profiles of mental health outcomes for the UK samples are displayed in Additional file 1: Table S11, and Fig. 4 presents the outcomes for each profile. The results indicated that the sample in the “*Low-Maltreated*” profile exhibited better mental health outcomes, such as higher levels of self-esteem and lower levels of anxiety, depression, and aggression. Moreover, the “*Severe*-*Maltreated*” profile displayed poorer mental health outcomes, with the highest level of depression, anxiety, and aggression and the lowest level of self-esteem.

When compared on depression levels, the “*Low-Maltreated*” profile was categorised as showing minimal depression, “*Moderate-Maltreated*” and “*High-Maltreated*” were categorised as showing mild and moderate depression, while the “*Severe-Maltreated*” profile was categorised as showing moderately severe depression. The difference between profiles was significant, except for the “*High-Maltreated*” and “*Severe-Maltreated*” profiles. Only the “*Low-Maltreated*” profile was below the clinical cut-off point when compared to anxiety levels. Other profiles were all above the clinical cut-off point. Except for the difference between the “*Moderate-Maltreated*” and “*Severe*-*Maltreated*” profiles being non-significant, differences between other profiles were all significant.

Regarding anger scores, the “*Low-Maltreated*” profile was below the norm mean score, while other profiles were all above the norm mean score. Differences between “*Moderate-Maltreated*”, “*High-Maltreated*,” and “*Severe-Maltreated*” were non-significant, while the difference between the “*Low-Maltreated*” profile and other profiles was significant. When compared to physical aggression, all the profiles were below the norm mean score. Like anger, the difference between the “*Low-Maltreated*” and other profiles was significant, while the differences between the other three profiles were non-significant. When compared to verbal aggression scores, all profiles were above the norm mean scores except the “*Low-Maltreated*” profile. Differences between all profiles were non-significant except for the difference between the “*Low-Maltreated*” and “*Moderate-Maltreated*” profiles. Compared to hostility scores, all the profiles were above the norm mean scores. Differences between all profiles were significant.

All the profiles were within the normal range when compared to self-esteem scores. The differences between “*Moderate-Maltreated*” and “*High-Maltreated*” and “*High-Maltreated*” and “*Severe-Maltreated*” profiles were non-significant. Other profiles showed significant differences from each other.

## Discussion

The current study aimed to identify latent profiles of childhood psychological maltreatment experiences in a Chinese and UK sample and explore which profiles were associated with poorer mental health outcomes. Results from the Chinese sample suggested a four-profile optimal solution for childhood psychological maltreatment comprising the profiles: "*Psychological Non-support*", ‘‘*Low-Maltreated*’’, ‘‘*High-Maltreated*’’, and ‘‘*Severe-Maltreated’’*. Results from the UK sample supported a four-profile solution for childhood psychological maltreatment, but the profiles differed from those in the Chinese sample. The profiles were: ‘‘*Low-Maltreated*’’, ‘‘*Moderate-Maltreated*’’, ‘‘*High-Maltreated*’’, and "*Severe-Maltreated*". Individuals in the "*Severe-Maltreated*" profile in China and the UK demonstrated the worst mental health outcomes, including higher levels of depression, anxiety, aggression, and lower self-esteem. In addition, individuals in the ‘‘*Psychological’’ Non-support*’’ profile in the Chinese sample and the "*Low-Maltreated*" profiles in the UK sample showed better mental health status, for instance, lower levels of depression, anxiety, and aggression, with a higher level of self-esteem.

In the current study, the pattern of childhood psychological maltreatment differed between the Chinese and UK sample. In the UK sample, the four profiles of psychological maltreatment only differed in severity; however, in the Chinese sample, there was a “Psychological Non-support” profile characterised by a low level of psychological abuse and neglect but with a high level of psychological non-support. According to previous studies [39], in Chinese culture, caregivers are more likely to show their support through an implicit approach instead of a direct or explicit approach. This might explain the low level of psychological abuse and neglect accompanied by higher psychological non-support in Chinese samples. Besides the “*Psychological Non-support*” profile, the other Chinese profiles only differed in severity, similar to the UK profiles. In addition, both samples had a similar severity pattern characterised by a clustering of different types of psychological maltreatment (i.e., higher psychological abuse, with higher psychological neglect). This means that children experiencing psychological abuse (or adults who experienced psychological abuse in childhood) may also be experiencing (or have experienced) psychological neglect, a possibility that clinicians or therapists should be aware of.

In addition, we found that the numbers of participants in the "*Low-Maltreated*" and "*Moderate-Maltreated*" in the UK sample were larger than in the Chinese sample. Indeed, a recent systematic review [63] demonstrated a high pooled prevalence of child maltreatment in China (physical abuse: 20%,emotional abuse: 30%; emotional neglect; 44%; sexual abuse: 12%; physical neglect: 47%). Another review [31] explored the prevalence of child maltreatment in the UK and suggested that the prevalence is somewhat lower in this context, with physical abuse, emotional abuse, sexual abuse, and neglect prevalence rates of approximately 18.9%, 15.6%, 8.7%, and 5.7% respectively. A possible reason for this is that Chinese cultures emphasise an adult-centric perspective instead of a child-centric perspective, and a cited Chinese proverb states that 'Beating and scolding is the emblem of love'. Some verbal or physical aggression may thus be viewed as ‘discipline’ or parenting rather than maltreatment by their caregivers [40]. Further, verbal aggression (e.g., scolding—a form of psychological abuse–may be more culturally accepted in this cultural context. Taken together, this may explain the observation that the scores of childhood psychological maltreatment were relatively higher than in the UK,however, the level of depression and anxiety was lower than in the UK samples.

The current study found that all the profiles (in both Chinese and the UK) had higher scores in the psychological abuse sub-dimension compared to the others, except the "*Severe-Maltreated*" profiles in the UK population, in which the psychological neglect levels were higher than the psychological abuse and psychological non-support levels. The results suggested that individuals with this profile had the highest level of depression, anxiety, and aggression, with the lowest level of self-esteem. The prevalence of psychological neglect was high and significantly related to mental health. Taillieu et al. [61] investigated the data from the National Epidemiological Survey and found that the most prevalent child maltreatment was psychological neglect (6.2%). They also found that psychological neglect was significantly related to personality disorders such as antisocial personality disorder, mood disorders such as major depression, and anxiety disorders such as generalised anxiety disorder. These findings are in line with the recent study, giving a potential explanation that individuals within "*Severe-Maltreated*" had the poorest level of mental health.

Moreover, the results also suggested that in Chinese and the UK profiles, the “*High-Maltreated*” and “*Severe-Maltreated*” profiles did not have significant differences on multiple tests, which may be able to explain by the plateau effect. Previous studies suggested that the plateau effect was seen beyond two adverse childhood experiences [15], some suggested that there was a plateau effect for the risky behaviours after three or more adverse childhood experiences [16]. Our study provides an insight that different forms (i.e., psychological abuse, neglect, and non-support) of a specific child maltreatment (i.e., psychological maltreatment) might have the plateau effect.

This study offered a novel investigation into childhood psychological maltreatment using LPA, a person-centric research strategy. Most of the past research using LPA has focused on different childhood adversity experiences [21]. The majority of other research in this field has relied upon variable-centric research approaches such as correlation or multiple regression and the associations between a focal adverse childhood experience and an outcome of interests [24]. In contrast, the person-centric research approach (i.e., latent profile analyses) provides a means to examine different forms of childhood psychological maltreatment as they cluster. By taking a more integrated perspective of childhood psychological maltreatment and acknowledging the potential interdependence between different forms of psychological maltreatment, it is possible to characterise and investigate the impact of the co-occurrence of different forms of psychological maltreatment. It can, for example, provide insights into the combined effect of childhood psychological maltreatment on mental health without the assuming additive effects of different types of psychological maltreatment.

Previous research has suggested that childhood psychological maltreatment is associated with long-term detrimental effects on adult mental health, including major depression, anxiety, suicidality, non-suicidal self-injury, substance abuse, and personality disorder (see [65, 66] for review). The current study results contribute to the growing evidence for the impacts of childhood psychological maltreatment on adult mental health. They thus underline the importance of preventing psychological maltreatment and providing mental health support to those affected. However, they also illuminate the impact of combinations of experiences of childhood psychological maltreatment on depression, anxiety, self-esteem, and aggression. Here we found, for example, that while three of the classes that emerged from the analysis in the Chinese sample differed primarily on the severity of psychological maltreatment, one class differed in its pattern from the others and was characterized primarily by a lack of psychological support. This class had elevated levels of issues relative to the low maltreatment class on a range of outcomes pointing to the fact that it is not only psychological neglect and abuse that may be harmful to psychosocial development but also a lack of psychological support occurring in the absence of any abuse. As such, interventions such as parenting programmes should take a broader focus to encompass all forms of psychological maltreatment (i.e., psychological abuse, psychological neglect, and psychological non-support) prevention and ensure that they equip parents to provide practical psychological support.

The primary limitations of our study relate to measurement. Self-report measurements may have some biases; however, previous evidence suggests that they provide valuable information. Hardt and Rutter [32] reviewed the validity of retrospective reports of childhood adversity and suggested that there were false-negative and substantial measurement errors. These errors or biases were not significant enough to invalidate retrospective measurement, though. However, another recent meta-analysis on the agreement between prospective and retrospective measures of childhood maltreatment [8] found that the agreement between prospective and retrospective reports of childhood maltreatment was poor. In addition, compared to the questionnaire, the agreement was higher when retrospective reports were based on interviews. Reuben et al. [50] found that retrospective adverse childhood experiences measurements were robust and associated with subjective life outcomes (e.g., physical health, cognitive impairment, general psychopathology, poor partner relationship quality),however, the agreement between prospective and retrospective emotional abuse was poor. Given the challenges of gathering prospective data, retrospective measurements of childhood adversity still have an important place in research. Nevertheless, it will be important for future studies to examine childhood psychological maltreatment profiles and their mental health outcomes using alternative measurement approaches to examine the robustness of findings across different methods.

Another limitation is the absence of age of onset of childhood psychological maltreatment. We did not measure during which developmental stages or ages the sample were exposed to psychological maltreatment in the current study because it was a retrospective study, and we did not expect that participants would be able to recall this accurately. However, previous studies have suggested that the age of onset of child maltreatment matters. For instance, Kaplow and Widom [35] found that earlier onset of physical and sexual abuse and neglect predicted more depression and anxiety symptoms, while the later onset of maltreatment predicted more behavioural problems in adulthood. Dunn et al. [23] similarly found that exposure to maltreatment during early childhood was most strongly associated with depression. Moreover, emotional neglect at ages 4–5 was found to be related to increased symptoms of dissociation, while emotional neglect at 8–9 enhanced symptoms of depression Schalinski et al. [56]. This suggests that the timing and/or chronicity of exposure to child abuse during childhood influences later mental health outcomes. For future studies, researchers should investigate exposure to psychological maltreatment by caregivers during different periods of childhood.

## Conclusion

The profiles of the Chinese and the UK suggest country context differences in psychological maltreatment profiles. The current paper suggested four profiles for both the Chinese and the UK populations. For the Chinese population, the profiles are divided into “*Psychological Non-support*”, “*Low-Maltreated*”, “*High-Maltreated*”, and “*Severe-Maltreated*”. For the UK population, the profiles are divided into “*Low-Maltreated*”, “*High-Maltreated*”, “Moderate-Maltreated”, and “*Severe-Maltreated*”. In both populations, individuals within the “Severe-Maltreated” profiles had the poorest mental health outcomes, with the highest depression, anxiety, and aggression level and the lowest level of self-esteem.

Childhood psychological maltreatment is associated with adult mental health outcomes. Taking a newer approach to examining the profiles and configuration of psychological maltreatment, the current study explored psychological abuse, psychological neglect, and psychological non-support and their joint associations with adult mental health demonstrating the associations between combined forms of psychological maltreatment and mental health outcomes. The LPA approach reveals a difference in patterns between China and the UK, which would not have been evident if applied variable-centric approach. This study underscores the importance of using targeted intervention or prevention to prevent psychological maltreatment and improve mental health outcomes in individuals with a history of psychological maltreatment. It also underlines the importance of equipping parents to provide practical psychological support as a lack of psychological support, even in the absence of psychological abuse and neglect, may be associated with poorer mental health and wellbeing outcomes.

## Supplementary Information

**Additional file 1: Table S1.** Demographic Characteristics (China). **Table S2.** Demographic Characteristics (UK). **Table S3.** Cronbach’s Alpha for all measures in both China and the UK sample. **Table S4.** Correlations between variables (China). Table S5. Correlations between variables (UK). **Table S6.** Fit statistic from the latent profile analysis model (China). **Table S7.** Fit statistic from the latent profile analysis model (UK). **Table S8.** Selected 4-profile Model in the whole sample (China). Table S9. Selected 4-profile Model in the whole sample (UK). **Table S10.** Relations of the 4-profile model on mental health outcomes (China). **Table S11.** Relations of the 4-profile model on mental health outcomes (UK). **Table S12.** Fit statistic from the latent profile analysis model (China). **Table S13.** Relations of the 4-profile model on mental health outcomes (China). **Table S14.** Fit statistic from the latent profile analysis model (UK). **Table S15.** Relations of the 4-profile model on mental health outcomes (UK)

## Data Availability

Additional Materials are available via https://osf.io/nec7a/?view_only=1e634bc71b86469ea8f149fa0c227065
