# Supplementary material for: Latent profiles of childhood psychological maltreatment and their links to adult mental health in China and the UK
Source: Child Adolesc Psychiatry Ment Health. 2023 Feb 24;17:30. doi: 10.1186/s13034-023-00572-4 (PMC9960471; doi:10.1186/s13034-023-00572-4)
Supplement: Supplementary file 1 — Additional file 1: Table S1. Demographic Characteristics (China). Table S2. Demographic Characteristics (UK). Table S3. Cronbach’s Alpha for all measures in both China and the UK sample. Table S4. Correlations between variables (China). Table S5. Correlations between variables (UK). Table S6. Fit statistic from the latent profile analysis model (China). Table S7. Fit statistic from the latent profile analysis model (UK). Table S8. Selected 4-profile Model in the whole sample (China). Table S9. Selected 4-profile Model in the whole sample (UK). Table S10. Relations of the 4-profile model on mental health outcomes (China). Table S11. Relations of the 4-profile model on mental health outcomes (UK). Table S12. Fit statistic from the latent profile analysis model (China). Table S13. Relations of the 4-profile model on mental health outcomes (China). Table S14. Fit statistic from the latent profile analysis model (UK). Table S15. Relations of the 4-profile model on mental health outcomes (UK) [file 13034_2023_572_MOESM1_ESM.docx]

**Supplementary Material**

Table S1. Demographic Characteristics (China)

Table S2. Demographic Characteristics (UK)

Table S3. Cronbach’s Alpha for all measures in both China and the UK sample

Table S4. Correlations between variables (China)

Table S5. Correlations between variables (UK)

Table S6. Fit statistic from the latent profile analysis model (China)

Table S7. Fit statistic from the latent profile analysis model (UK)

Table S8. Selected 4-profile Model in the whole sample (China)

Table S9. Selected 4-profile Model in the whole sample (UK)

Table S10. Relations of the 4-profile model on mental health outcomes (China)

Table S11. Relations of the 4-profile model on mental health outcomes (UK)

Supplementary Material 2. Sensitivity Analysis

| Table S1. Demographic Characteristics (China) | | | |
| --- | --- | --- | --- |
|  |  | N | % |
| Gender | Female | 346 | 63.4% |
|  | Male | 197 | 36.1% |
|  | Non-binary | 3 | 0.5% |
| Age | 18-20 | 114 | 20.9% |
|  | 21-30 | 327 | 59.9% |
|  | 31-40 | 90 | 16.5% |
|  | 41-60 | 14 | 2.6% |
|  | Over 60 | 1 | 0.2% |
| Primary Caregiver | Mother | 337 | 61.6% |
|  | Father | 98 | 17.9% |
|  | Stepmother | 3 | 0.5% |
|  | Stepfather | 1 | 0.2% |
|  | Grandparents | 73 | 13.3% |
|  | Nanny | 6 | 1.1% |
|  | Others | 29 | 5.3% |
| Education Level | Below high school | 7 | 1.3% |
|  | High school | 49 | 9.0% |
|  | Undergraduate | 371 | 67.8% |
|  | Postgraduate | 105 | 19.2% |
|  | PhD | 15 | 2.7% |
| Notes. N = 544. Others refer to other relatives such as uncle or aunt. | | | |

| Table S2. Demographic Characteristics (UK) | | | |
| --- | --- | --- | --- |
|  |  | N | % |
| Gender | Female | 371 | 63.0% |
|  | Male | 208 | 35.3% |
|  | Non-binary | 10 | 1.70% |
| Age | 18-20 | 357 | 60.6% |
|  | 21-30 | 171 | 29.0% |
|  | 31-40 | 47 | 8.0% |
|  | 41-60 | 13 | 2.2% |
|  | Over 60 | 1 | 0.2% |
| Primary Caregiver | Mother | 498 | 84.2% |
|  | Father | 43 | 7.3% |
|  | Grandparents | 26 | 4.4% |
|  | Nanny | 7 | 1.2% |
|  | Others | 17 | 29.% |
| Education Level | Below high school | 3 | 0.5% |
|  | High school | 111 | 18.8% |
|  | Undergraduate | 396 | 67.2% |
|  | Postgraduate | 65 | 11.0% |
|  | PhD | 14 | 2.4% |
| Notes. N = 589. Others refer to others refer to domestic helper, other relative such as great grandparent. | | | |

| Table S3. Cronbach’s Alpha for all measures in both China and the UK sample | | |
| --- | --- | --- |
|  | China | UK |
| PA | 0.89 | 0.90 |
| PN | 0.90 | 0.94 |
| PS | 0.84 | 0.89 |
| ACE | 0.74 | 0.58 |
| Anxiety | 0.91 | 0.94 |
| Depression | 0.87 | 0.89 |
| Self-esteem | 0.85 | 0.82 |
| Physical aggression | 0.73 | 0.82 |
| Verbal aggression | 0.58 | 0.75 |
| Anger | 0.76 | 0.81 |
| Hostility | 0.82 | 0.78 |

| Table S4. Correlations between variables (China) | | | | | | | | | | |
| --- | --- | --- | --- | --- | --- | --- | --- | --- | --- | --- |
|  | PA | PN | PNS | Self-esteem | Anxiety | Anger | Physical Aggression | Hostility | Verbal Aggression | depression |
| PA | 1 | .76 | .24 | -.265 | .36 | .37 | .39 | .35 | .30 | .42 |
| PN | <.001 | 1 | .41 | -.29 | .44 | .32 | .37 | .35 | .24 | .44 |
| PNS | <.001 | <.001 | 1 | -.26 | .31 | .05 | .05 | .04 | -.27 | .13 |
| Self-esteem | <.001 | <.001 | <.001 | 1 | -.55 | -.33 | -.27 | -.46 | -.09 | -.57 |
| Anxiety | <.001 | <.001 | <.001 | <.001 | 1 | .50 | .47 | .47 | .10 | .70 |
| Anger | <.001 | <.001 | .250 | <.001 | <.001 | 1 | .70 | .62 | .47 | .45 |
| Physical Aggression | <.001 | <.001 | .398 | <.001 | <.001 | <.001 | 1 | .56 | .50 | .43 |
| Hostility | <.001 | <.001 | .271 | <.001 | <.001 | <.001 | <.001 | 1 | .56 | .54 |
| Verbal Aggression | <.001 | <.001 | <.001 | .039 | .024 | <.001 | <.001 | <.001 | 1 | .30 |
| Depression | <.001 | <.001 | .004 | <.001 | <.001 | <.001 | <.001 | <.001 | <.001 | 1 |
| Notes. PA = Psychological Abuse, PN = Psychological Neglect, PNS = Psychological Non-Support. Pearson correlations above the diagonal; *p­­*-values below the diagonal. | | | | | | | | | | |

| Table S5. Correlations between variables (UK) | | | | | | | | | | |
| --- | --- | --- | --- | --- | --- | --- | --- | --- | --- | --- |
|  | PA | PN | PNS | Self-esteem | Anxiety | Anger | Physical Aggression | Hostility | Verbal Aggression | Depression |
| PA | 1 | .79 | .46 | -.34 | .38 | .27 | .20 | .39 | .12 | .41 |
| PN | <.001 | 1 | .57 | -.39 | .45 | .29 | .20 | .42 | .11 | .41 |
| PNS | <.001 | <.001 | 1 | -.32 | .28 | .20 | .18 | .28 | .10 | .26 |
| Self-esteem | <.001 | <.001 | <.001 | 1 | -.58 | -.38 | -.21 | -.57 | -.11 | -.58 |
| Anxiety | <.001 | <.001 | <.001 | <.001 | 1 | .35 | .15 | .54 | .06 | .69 |
| Anger | <.001 | <.001 | <.001 | <.001 | <.001 | 1 | .60 | .48 | .55 | .32 |
| Physical Aggression | <.001 | <.001 | <.001 | <.001 | <.001 | <.001 | 1 | .38 | .43 | .16 |
| Hostility | <.001 | <.001 | <.001 | <.001 | <.001 | <.001 | <.001 | 1 | .28 | .53 |
| Verbal Aggression | .005 | .010 | .016 | .011 | .141 | <.001 | <.001 | <.001 | 1 | .15 |
| Depression | <.001 | <.001 | <.001 | <.001 | <.001 | <.001 | <.001 | <.001 | <.001 | 1 |
| Notes. PA = Psychological Abuse, PN = Psychological Neglect, PNS = Psychological Non-Support. Pearson correlations above the diagonal; *p­­*-values below the diagonal. | | | | | | | | | | |

| Table S6. Fit statistic from the latent profile analysis model (China) | | | | | | |
| --- | --- | --- | --- | --- | --- | --- |
|  | LMR | p | AIC | BIC | aBIC | Entropy |
| Class 1 | / | / | 10667.310 | 10701.701 | 10676.306 | / |
| Class 2 | 378.032 | <.001 | 10086.973 | 10133.659 | 10098.743 | 0.827 |
| Class 3 | 139.353 | 0.0402 | 9953.157 | 10021.064 | 9970.277 | 0.851 |
| **Class 4** | **81.934** | **0.0002** | **9878.599** | **9967.726** | **9901.068** | **0.860** |
| Class 5 | 51.146 | 0.2112 | 9835.815 | 9946.163 | 9863.634 | 0.861 |
| Class 6 | 55.911 | 0.2532 | 9788.411 | 9919.980 | 9821.581 | 0.844 |
| Class 7 | 37.515 | 0.6135 | 9760.037 | 9912.827 | 9798.557 | 0.857 |
| Class 8 | 36.081 | 0.0689 | 9733.234 | 9907.245 | 9777.104 | 0.870 |
| *Notes.* LMR = Lo-Mendell-Rubin likelihood ratio test; AIC = Akaike’s Information Criteria; BIC = Bayesian Information Criteria; aBIC = adjusted Bayesian Information Criteria. ACE as a covariate. | | | | | | |

| Table S7. Fit statistic from the latent profile analysis model (UK) | | | | | | |
| --- | --- | --- | --- | --- | --- | --- |
|  | LMR | p | AIC | BIC | aBIC | Entropy |
| Class 1 | / | / | 22108.657 | 22143.684 | 22118.287 | / |
| Class 2 | 835.845 | <.001 | 12753.071 | 12801.233 | 12766.312 | 0.941 |
| Class 3 | 264.390 | 0.0909 | 12490.391 | 12560.446 | 12509.651 | 0.888 |
| **Class 4** | **187.693** | **0.0000** | **12306.812** | **12398.759** | **12332.091** | **0.911** |
| Class 5 | 74.360 | 0.1277 | 12240.121 | 12353.960 | 12271.418 | 0.899 |
| Class 6 | 58.579 | 0.4172 | 12189.705 | 12325.436 | 12227.022 | 0.908 |
| Class 7 | 54.347 | 0.1215 | 12143.654 | 12301.277 | 12186.989 | 0.909 |
| Class 8 | 25.951 | 0.8156 | 12126.889 | 12306.404 | 12176.243 | 0.876 |
| *Notes.* LMR = Lo-Mendell-Rubin likelihood ratio test; AIC = Akaike’s Information Criteria; BIC = Bayesian Information Criteria; aBIC = adjusted Bayesian Information Criteria. ACE as a covariate. | | | | | | |

| Table S8. Selected 4-profile Model in the whole sample (China) | | | | | | | |
| --- | --- | --- | --- | --- | --- | --- | --- |
| Class | Class Size* | Psychological Abuse | | Psychological Neglect | | Psychological Non-support | |
|  |  | M | SE | M | SE | M | SE |
| Psychological Non-support | 5.4% | 4.79 | 1.29 | 4.24 | 1.10 | 42.30 | 1.57 |
| Low-Maltreated | 56% | 11.96 | 0.69 | 7.61 | 0.50 | 11.76 | 0.67 |
| High-Maltreated | 32.9% | 28.35 | 1.47 | 24.75 | 1.52 | 23.24 | 0.62 |
| Severe-Maltreated | 5.5% | 46.93 | 4.01 | 44.90 | 3.65 | 30.83 | 4.18 |
| *Notes.* *Based on estimated posterior probabilities. | | | | | | | |

| Table S9. Selected 4-profile Model in the whole sample (UK) | | | | | | | |
| --- | --- | --- | --- | --- | --- | --- | --- |
| Class | Class Size* | Psychological Abuse | | Psychological Neglect | | Psychological Non-support | |
|  |  | M | SE | M | SE | M | SE |
| Low-Maltreated | 57.9% | 8.935 | 0.474 | 3.700 | 0.236 | 17.104 | 0.343 |
| Moderate-Maltreated | 25.4% | 21.909 | 1.192 | 14.931 | 0.831 | 24.081 | 0.683 |
| High-Maltreated | 11.2% | 32.119 | 1.348 | 31.774 | 1.355 | 27.764 | 1.034 |
| Severe-Maltreated | 5.5% | 47.753 | 1.376 | 51.483 | 1.168 | 30.619 | 1.566 |
| *Notes.* *Based on estimated posterior probabilities. | | | | | | | |

| Table S10. Relations of the 4-profile model on mental health outcomes (China) | | | | | | | | | | | | |  |  |
| --- | --- | --- | --- | --- | --- | --- | --- | --- | --- | --- | --- | --- | --- | --- |
| Outcome means (SE) by class | | | | | | | | | | | | | | |
|  | Psychological Non-Support | | Low-Maltreated | | | High-Maltreated | | Severe-Maltreated | | |  |  | |  |
| Self-esteem | 31.38 (.95) | | 30.70 (.31) | | | 27.74 (.41) | | 24.78 (1.14) | |  | |  | |  |
| Anxiety | 30.31 (1.90) | | 24.38 (.82) | | | 40.51 (1.17) | | 41.05 (4.04) | |  | |  | |  |
| Depression | 2.63 (.69) | | 5.615 (.29) | | | 9.84 (.41) | | 11.05 (1.51) | |  | |  | |  |
| Anger | 14.47 (.70) | | 17.67 (0.33) | | | 20.19 (.37) | | 23.50 (1.28) | |  | |  | |  |
| Physical aggression | 17.51 (1.03) | | 21.67 (.34) | | | 25.30 (.41) | | 27.33 (1.67) | |  | |  | |  |
| Verbal aggression | 8.97 (.79) | | 15.05 (.18) | | | 15.44 (.22) | | 16.05 (.96) | |  | |  | |  |
| Hostility | 13.63 (1.19) | | 22.35 (.36) | | | 24.42 (.40) | | 28.80 (.98) | |  | |  | |  |
| **Wald test *p* value** | C1 vs. C2 | C1 vs. C3 | | C1 vs. C4 | C2 vs. C3 | | C2 vs. C4 | | C3 vs. C4 | | | |  |  |
| Self-esteem | 0.496 | <.001** | | <.001** | <.001** | | <.001** | | 0.016 | | | |  |  |
| Anxiety | 0.005* | <.001** | | 0.003* | <.001** | | <.001** | | 0.477 | | | |  |  |
| Depression | <.001** | <.001** | | <.001** | <.001** | | <.001** | | 0.151 | | | |  |  |
| Anger | <.001** | <.001** | | <.001** | <.001** | | <.001** | | 0.001* | | | |  |  |
| Physical aggression | <.001** | <.001** | | <.001** | <.001** | | <.001** | | 0.049 | | | |  |  |
| Verbal aggression | <.001** | <.001** | | <.001** | 0.529 | | 0.066 | | 0.112 | | | |  |  |
| Hostility | <.001** | <.001** | | <.001** | 0.002* | | <.001** | | <.001** | | | |  |  |
| *Notes.* C1 = Psychological Non-support; C2 = Low-Maltreated; C3 = High-Maltreated; C4 = Severe-Maltreated. According to Bonferroni correction, the new p value = .0083. p <.0083*, p <.001**. | | | | | | | | | | | | |  |  |

| Table S11. Relations of the 4-profile model on mental health outcomes (UK) | | | | | | |
| --- | --- | --- | --- | --- | --- | --- |
| Outcome means (SE) by class | | | | |  |  |
|  | Low-Maltreated | Moderate-Maltreated | High-Maltreated | Severe-Maltreated |  |  |
| Self-esteem | 29.95 (.27) | 26.46 (.45) | 25.23 (.63) | 23.82 (.95) |  |  |
| Anxiety | 24.16 (.89) | 34.30 (1.44) | 44.61 (2.26) | 50.07 (3.54) |  |  |
| Depression | 8.10 (.34) | 11.40 (.52) | 15.29 (.80) | 16.90 (1.30) |  |  |
| Anger | 14.75 (.31) | 17.61 (.51) | 18.69 (.78) | 19.88 (1.31) |  |  |
| Physical aggression | 16.77 (.35) | 20.42 (.62) | 20.27 (.93) | 20.38 (1.86) |  |  |
| Verbal aggression | 13.60 (.24) | 15.00 (.42) | 14.70 (.57) | 15.41 (.95) |  |  |
| Hostility | 20.12 (.36) | 24.54 (.49) | 26.19 (.71) | 29.75 (.90) |  |  |
| **Wald test *p* value** | C1 vs. C2 | C1 vs. C3 | C1 vs. C4 | C2 vs. C3 | C2 vs. C4 | C3 vs. C4 |
| Self-esteem | <.001** | <.001** | <.001** | 0.122 | 0.012 | 0.225 |
| Anxiety | <.001** | <.001** | <.001** | <.001** | <.001** | 0.20 |
| Depression | <.001** | <.001** | <.001** | <.001** | <.001** | 0.302 |
| Anger | <.001** | <.001** | <.001** | 0.263 | 0.108 | 0.443 |
| Physical aggression | <.001** | <.001** | <.001** | 0.896 | 0.983 | 0.960 |
| Verbal aggression | 0.005* | 0.072 | 0.065 | 0.687 | 0.690 | 0.529 |
| Hostility | <.001** | <.001** | <.001** | 0.062 | <.001** | 0.002* |
| *Notes.* C1 = Low Maltreatment; C2 = Moderate-Maltreated; C3 = High-Maltreated; C4 = Severe-Maltreated. According to Bonferroni correction, the new p value = .0083. p <.0083*, p <.001**. | | | | | | |

**Supplementary Material**

**Latent Profile Solution – Chinese Samples**

A series of latent profile models with one to eight were specified and estimated. The LMR test had a non-significant value for the 6-class model (*p* > .05), suggesting a 5-class optimal model. However, the p-value of the 5-class model LMR test was close to our *p*-threshold of .05 and we therefore also considered the 4-class model as a further candidate optimal model. Other than the LMR test, we also utilized criteria for AIC, BIC, and aBIC for model selection. These suggested that the five-profile solution should be preferred. However, Foti et al. (2012) highlighted that the meaning of the profile-solution picked needs to be considered when determining the number of profiles. We thus further investigated the characteristics of these solutions. We found that choosing a four-profile solution translated into the addition of a meaningful profile, while the five-profile solution shared similar characteristics with the four-profile solution (see Table S12).

Based on the aforementioned rationales, we thus explored further the four-profile model in the Chinese sample. Table S12 indicated that the entropy of the four-profile solution was 0.904, which is considered satisfactory (Morin et al., 2011). Additionally, the average latent class probabilities for the four-profile model were 0.959, 0.928, 0.937, and 0.919, above the cut-off criterion of 0.80 (Nylund, Asparouhov, & Muthén, 2007). Accordingly, we adopted the four-profile model for the Chinese samples as the best solution based on theoretical and statistical considerations. The first latent profile was the smallest and described the 5.4% of the sample who reported the lowest level of psychological abuse and psychological neglect but the highest level of psychological non-support and was thus labelled the “Psychological Non-support” profile. The second latent profile represented 56.5% of the samples was labeled “Low-Maltreated,” given that the level of psychological abuse and psychological neglect was higher than the “Psychological Non-support” group, but with the lowest level of psychological non-support. The third latent profile, named the “High-Maltreated” profile, characterized 33.9% of the sample presenting experiences of a higher level of psychological abuse, psychological neglect, and psychological non-support. The fourth latent profile, labeled “*Severe*-Maltreated”, includes 4.3% of the sample and reported the highest level of psychological abuse, psychological neglect, and psychological non-support. Table S13 presents the means and standard errors of each psychological maltreatment type.

**Latent Profile Solution – UK Samples**

The four-class solution was also considered the best fitting model for the UK sample based on a series of fit indices (see Table S14). The LMR test had a non-significant value for the 5-class model (p > .05), suggesting a 4-class optimal model and the BIC, which has been identified as the most reliable of the available fit indices (Nylund et al., 2007), was lower than other models with significant *p*-values. We, therefore, adopted the four-profile model as the best solution for further analyses.

The first latent profile was the largest and described 57.9% of the sample who reported the lowest level of childhood psychological maltreatment and were thus “Low-Maltreated”. The second latent profile represented by 25.4% of the sample was labeled “Moderate-Maltreated,” given that samples experienced a moderate level of childhood psychological maltreatment. The third latent profile, named the “High-Maltreated” profile, characterized 11.2% of samples, representing a higher level of childhood psychological maltreatment. The fourth latent profile, labeled “Severe-Maltreated”, included 5.5% of the sample who reported the highest level of childhood psychological maltreatment. Table S15 presents the means and standard error for each form of psychological maltreatment.

**Reference**

Foti, R. J., Bray, B. C., Thompson, N. J., & Allgood, S. F. (2012). Know thy self, know thy leader: Contributions of a pattern-oriented approach to examining leader perceptions. *The Leadership Quarterly*, *23*(4), 702-717.

Morin, A. J., Maiano, C., Nagengast, B., Marsh, H. W., Morizot, J., & Janosz, M. (2011). General growth mixture analysis of adolescents' developmental trajectories of anxiety: The impact of untested invariance assumptions on substantive interpretations. *Structural Equation Modeling: A Multidisciplinary Journal*, *18*(4), 613-648.

Nylund, K. L., Asparouhov, T., & Muthén, B. O. (2007). Deciding on the number of classes in latent class analysis and growth mixture modeling: A Monte Carlo simulation study. *Structural equation modeling: A multidisciplinary Journal*, *14*(4), 535-569.

| Table S12. Fit statistic from the latent profile analysis model (China) | | | | | | |
| --- | --- | --- | --- | --- | --- | --- |
|  | LMR | p | AIC | BIC | aBIC | Entropy |
| Class 1 | / | / | 12863.678 | 12889.472 | 12870.425 | / |
| Class 2 | 436.920 | <.001 | 12417.417 | 12460.407 | 12428.663 | 0.838 |
| Class 3 | 150.501 | 0.0066 | 12268.942 | 12329.128 | 12284.686 | 0.885 |
| **Class 4** | **137.151** | **<.001** | **12134.348** | **12211.729** | **12154.590** | **0.904** |
| Class 5 | 91.642 | 0.0240 | 12047.068 | 12141.645 | 12071.809 | 0.863 |
| Class 6 | 67.190 | 0.2939 | 11985.211 | 12096.984 | 12014.450 | 0.868 |
| Class 7 | 63.947 | 0.0032 | 11926.726 | 12055.695 | 11960.463 | 0.859 |
| Class 8 | 22.772 | 0.5246 | 11911.051 | 12057.215 | 11949.286 | 0.868 |
| *Notes.* LMR = Lo-Mendell-Rubin likelihood ratio test; AIC = Akaike’s Information Criteria; BIC = Bayesian Information Criteria; aBIC = adjusted Bayesian Information Criteria. | | | | | | |

| Table S13. Relations of the 4-profile model on mental health outcomes (China) | | | | | | | | | | | | |  |  |
| --- | --- | --- | --- | --- | --- | --- | --- | --- | --- | --- | --- | --- | --- | --- |
| Outcome means (SE) by class | | | | | | | | | | | | | | |
|  | Psychological Non-Support | | Low-Maltreated | | | High-Maltreated | | Severe-Maltreated | | |  |  | |  |
| Self-esteem | 31.37 (.94) | | 30.64 (.30) | | | 27.74 (.41) | | 24.78 (1.14) | |  | |  | |  |
| Anxiety | 30.65 (1.91) | | 24.38 (.82) | | | 40.51 (1.17) | | 41.05 (4.04) | |  | |  | |  |
| Depression | 2.655 (.69) | | 5.615 (.29) | | | 9.84 (.41) | | 11.05 (1.51) | |  | |  | |  |
| Anger | 14.47 (.71) | | 17.67 (0.33) | | | 20.19 (.37) | | 23.50 (1.28) | |  | |  | |  |
| Physical aggression | 17.69 (1.07) | | 21.67 (.34) | | | 25.30 (.41) | | 27.33 (1.67) | |  | |  | |  |
| Verbal aggression | 9.10 (.83) | | 15.05 (.18) | | | 15.44 (.22) | | 16.05 (.96) | |  | |  | |  |
| Hostility | 13.72 (1.23) | | 22.35 (.36) | | | 24.42 (.40) | | 28.80 (.98) | |  | |  | |  |
| **Wald test *p* value** | C1 vs. C2 | C1 vs. C3 | | C1 vs. C4 | C2 vs. C3 | | C2 vs. C4 | | C3 vs. C4 | | | |  |  |
| Self-esteem | 0.467 | <.001*** | | <.001*** | <.001*** | | <.001*** | | 0.016* | | | |  |  |
| Anxiety | 0.003** | <.001*** | | 0.020* | <.001*** | | <.001*** | | 0.901 | | | |  |  |
| Depression | <.001*** | <.001*** | | <.001*** | <.001*** | | <.001*** | | 0.446 | | | |  |  |
| Anger | <.001*** | <.001*** | | <.001*** | <.001*** | | <.001*** | | 0.015* | | | |  |  |
| Physical aggression | <.001*** | <.001*** | | <.001*** | <.001*** | | 0.001** | | 0.246 | | | |  |  |
| Verbal aggression | <.001*** | <.001*** | | <.001*** | 0.193 | | 0.304 | | 0.537 | | | |  |  |
| Hostility | <.001*** | <.001*** | | <.001*** | <.001*** | | <.001*** | | <.001*** | | | |  |  |
| *Notes.* C1 = Psychological Non-support; C2 = Low-Maltreated; C3 = High-Maltreated; C4 = Severe-Maltreated. | | | | | | | | | | | | |  |  |

| Table S14. Fit statistic from the latent profile analysis model (UK) | | | | | | |
| --- | --- | --- | --- | --- | --- | --- |
|  | LMR | p | AIC | BIC | aBIC | Entropy |
| Class 1 | / | / | 13605.126 | 13631.396 | 13612.348 | / |
| Class 2 | 741.295 | 0 | 12842.776 | 12886.56 | 12854.814 | 0.93 |
| Class 3 | 259.435 | 0.0633 | 12581.173 | 12642.471 | 12598.026 | 0.892 |
| **Class 4** | **165.918** | **0.0003** | **12416.752** | **12495.564** | **12438.42** | **0.908** |
| Class 5 | 74.146 | 0.3899 | 12347.7 | 12444.025 | 12374.183 | 0.892 |
| Class 6 | 57.508 | 0.5176 | 12295.937 | 12409.776 | 12327.235 | 0.903 |
| Class 7 | 52.99 | 0.168 | 12248.871 | 12380.224 | 12284.984 | 0.902 |
| Class 8 | 24.147 | 0.2828 | 12231.777 | 12380.644 | 12272.705 | 0.869 |
| *Notes.* LMR = Lo-Mendell-Rubin likelihood ratio test; AIC = Akaike’s Information Criteria; BIC = Bayesian Information Criteria; aBIC = adjusted Bayesian Information Criteria. | | | | | | |

| Table S15. Relations of the 4-profile model on mental health outcomes (UK) | | | | | | |
| --- | --- | --- | --- | --- | --- | --- |
| Outcome means (SE) by class | | | | |  |  |
|  | Low-Maltreated | Moderate-Maltreated | High-Maltreated | Severe-Maltreated |  |  |
| Self-esteem | 29.90 (.27) | 26.48 (.46) | 25.16 (.64) | 23.91 (.97) |  |  |
| Anxiety | 24.42 (.90) | 34.08 (1.46) | 44.98 (2.34) | 49.46 (3.57) |  |  |
| Depression | 8.12 (.33) | 11.54 (.54) | 15.27 (.81) | 16.74 (1.32) |  |  |
| Anger | 14.70 (.31) | 17.82 (.51) | 18.66 (.79) | 19.97 (1.34) |  |  |
| Physical aggression | 16.69 (.35) | 20.74 (.63) | 20.05 (.95) | 20.80 (1.85) |  |  |
| Verbal aggression | 13.53 (.24) | 15.21 (.42) | 14.72 (.58) | 15.34 (.97) |  |  |
| Hostility | 20.18 (.36) | 24.54 (.51) | 26.21 (.71) | 29.81 (.90) |  |  |
| **Wald test *p* value** | C1 vs. C2 | C1 vs. C3 | C1 vs. C4 | C2 vs. C3 | C2 vs. C4 | C3 vs. C4 |
| Self-esteem | <.001*** | <.001*** | <.001*** | 0.104 | 0.017* | 0.288 |
| Anxiety | <.001*** | <.001*** | <.001*** | <.001*** | <.001*** | 0.29 |
| Depression | <.001*** | <.001*** | <.001*** | <.001*** | <.001*** | 0.351 |
| Anger | <.001*** | <.001*** | <.001*** | 0.389 | 0.134 | 0.404 |
| Physical aggression | <.001*** | 0.001** | 0.029* | 0.559 | 0.974 | 0.721 |
| Verbal aggression | 0.001** | 0.054 | 0.07 | 0.509 | 0.898 | 0.589 |
| Hostility | <.001*** | <.001*** | <.001*** | 0.064 | <.001*** | 0.002** |
| *Notes.* C1 = Low Maltreatment; C2 = Moderate-Maltreated; C3 = High-Maltreated; C4 = Severe-Maltreated. | | | | | | |
